# Regular feedback on inter-hospital transfer improved the clinical outcome and survival in patients with multiple trauma: a retrospective cohort study

**DOI:** 10.1186/s12873-021-00543-y

**Published:** 2021-12-03

**Authors:** Chih-Jung Wang, Tsung-Han Yang, Kuo-Shu Hung, Chun-Hsien Wu, Shu-Ting Yen, Yi-Ting Yen, Yan-Shen Shan

**Affiliations:** 1 Division of Trauma, Department of Surgery, National Cheng Kung University Hospital, College of Medicine, National Cheng Kung University, No.138, Sheng Li Road, Tainan, Taiwan; 2 Division of General Surgery, Department of Surgery, National Cheng Kung University Hospital, Tainan, Taiwan; 3 Institute of Clinical Medicine, College of Medicine, National Cheng Kung University, Tainan, Taiwan

**Keywords:** Trauma, Transfer, Inter-hospital, Feedback, Quality

## Abstract

**Background:**

Undertriage of major trauma patients is unavoidable, especially in the trauma system of rural areas. Timely stabilization and transfer of critical trauma patients remains a great challenge for hospitals with limited resources. No definitive measure has been proven to improve the outcomes of patients transferred with major trauma. The current study hypothesized that regular feedback on inter-hospital transfer of patients with major trauma can improve quality of care and clinical outcomes.

**Method:**

This retrospective cohort study retrieved data of transferred major trauma patients with an injury severity score (ISS) > 15 between January 2010 and December 2018 from the trauma registry databank of a tertiary medical center. Regular monthly feedback on inter-hospital transfers was initiated in 2014. The patients were divided into a without-feedback group and a with-feedback group. Demographic data, management before transfer, and outcomes after transfer were collected and analyzed.

**Results:**

A total of 178 patients were included: 69 patients in the without-feedback group and 109 in the with-feedback group. The with-feedback group had a higher ISS (25 vs. 27; *p* = 0.049), more patients requiring massive transfusion (14.49% vs. 29.36%, *p* = 0.036), and less patients with Glasgow Coma Scale ≤8 (30.43% vs. 23.85%, *p* <  0.001). After adjusting for confounding factors, the with-feedback group was associated with a higher rate of blood transfusion before transfer (adjusted odds ratio [aOR]: 2.75; 95% confidence interval [CI]: 1.01–7.52; *p* = 0.049), shorter time span before blood transfusion (− 31.80 ± 15.14; *p* = 0.038), and marginally decreased mortality risk (aOR: 0.43; 95% CI: 0.17–1.09; *p* = 0.076).

**Conclusion:**

This study revealed that regular feedback on inter-hospital transfer improved the quality of blood transfusion.

## Background

Trauma injury is a global issue and will continue to be a major cause for mortality in the next decade [1, 2]. Trauma is one of the first medical specialties to develop regionalization, with criteria for transfer to dedicated trauma centers and the ability to track outcomes at these centers. A level I trauma center effectively decreases the mortality rate of trauma patients [3, 4]. Inclusion of regional trauma centers into the trauma system may facilitate timely transfer of severely injured patients and also decrease the overall mortality rate [3, 5–7]. Nonetheless, delayed transfer with inadequate initial resuscitation still poses a major issue, [8–10] and there is no room for improvement without feedback from the receiving hospital and specialists in trauma care.

Hospitals in Taiwan are not equally equipped with specialized trauma surgeons. Despite nationwide coverage of the health insurance system, patients who suffer major trauma in rural areas need to be resuscitated and then transferred to level I trauma centers. The accreditation of trauma and acute care systems started in 2010, and hospitals have been graded according to their capabilities in the management of patients with multiple traumas. Our hospital has been graded as a level 1 trauma center ever since and has been responsible for patients transferred from regional referral hospitals. During the last few years, peer review and feedback have become integral to medical education, improvement of skills, clinical judgment, and system development. We retrospectively analyzed the quality of resuscitation, clinical outcomes, and impact of regular feedback in multiple trauma patients referred from regional hospitals.

## Methods

We reviewed the critical major trauma patients at the weekly trauma conference. For the transferred major trauma patients, we also reviewed the pre-hospital care process including CT indication, diagnosis before transfer, resuscitation intensity, management, and time to transfer. All the suggestions and patient outcomes were relayed as feedback to the emergency department (ED) physicians of regional hospitals in the inter-hospital conference. This conference was attended by physicians and nurses from the regional hospital ED as well as the trauma surgeon and trauma manager from our hospital. Image interpretation, importance of blood transfusion, criteria for transfer, and treatment protocol were the common issues discussed in the conference. We do not have a regional trauma conference; the inter-hospital conference is the only way that the regional hospital and medical center can discuss how to improve the quality of care for major trauma patients who need transfer. Feedback on inter-hospital transfer was initiated in 2014 on a monthly basis, taking turns among the referring hospitals (about once every three months for each hospital). For the current study, patient characteristics and demographics were retrieved from the trauma registry database between January 2010 and December 2018. Adult (> 15 years) major trauma patients (injury severity score [ISS] > 15) transferred immediately after initial resuscitation for multiple trauma in regional hospitals in the study period were included; those staying longer than 12 h in regional hospitals were assumed to not be critically injured patients in need of immediate transfer and were excluded.

The inter-hospital transfer duration (IHTD) was defined as the time between the patient’s arrival at the ED of a regional hospital and our hospital. Patients’ need for transfusion was defined as transfusion of blood components after patient arrival at the ED of a regional hospital or our hospital. The time span before blood transfusion (TSBT) was defined as the duration between patient arrival at the ED of a regional hospital and transfusion of the first unit of packed red blood cells (PRBCs).

Continuous data were analyzed using the Mann-Whitney U test as appropriate, and categorized data were analyzed using the chi-squared test or Fisher’s exact test. Linear regression and logistic regression were performed as univariate and multivariate analyses for continuous and categorized outcomes, respectively.

## Results

There were 69 and 109 patients in the before-feedback and after-feedback groups, respectively. There was no difference in age or sex between the two groups. The with-feedback group had a higher ISS (25 vs. 27; *p* = 0.0499), more patients requiring transfusion of at least 12 units of PRBC (14.49% vs. 29.36%, *p* = 0.036), and less patients with Glasgow Coma Scale ≤8 (30.43% vs. 23.85%, *p* <  0.001). The with-feedback group had more patients undergoing cardiopulmonary resuscitation (one, 1.45% vs. eight, 7.34%), but the difference was not significant (*p* = 0.156). The ratio of patients with a shock index ≥0.9 in regional hospitals increased from 20.29% before feedback to 34.91% after feedback (*p* = 0.056). (Table 1).

**Table 1:** Patient demographics and outcomes

	Without feedback (*N* = 69)	With feedback (*N* = 109)	*p*-value ^a^
	n (%)	n (%)	
***Patient demographics***			
Age, median (IQR), years	41.0 (23.0–64.0)	46.0 (25.0–61.0)	0.800
Male	49 (71.01)	77 (70.64)	1.000
ISS, median (IQR)	25.0 (20.0–29.0)	27.0 (20.0–38.0)	0.0499
CPR	1 (1.45)	8 (7.34)	0.156
Shock index ≥0.9	14 (20.29)	37 (34.91)	0.056
GCS ≤ 8	21 (30.43)	26 (23.85)	< 0.001
RBC ≥12 U	10 (14.49)	32 (29.36)	0.036
***Outcome***			
IHTD, median (IQR), minutes	141.0 (107.0–184.0)	134.0 (103.0–170.0)	0.330
Patients with transfusion	*N* = 26	*N* = 71	
Blood transfusion before transfer	11 (42.31)	52 (73.24)	0.010
TSBT, median (IQR), minutes	101.5 (79.0–153.0)	88.0 (55.0–132.0)	0.046
Mortality	16 (23.19)	18 (16.51)	0.364
LOS, median (IQR), days	15.0 (8.0–22.0)	14.0 (9.0–29.0)	0.528

^a^Chi-squared test or Fisher’s exact test for categorical variables or the Mann-Whitney U test for continuous variables. ISS, injury severity score; CPR, cardiopulmonary resuscitation; GCS, Glasgow Coma Scale; RBC, red blood cell; IHTD, inter-hospital transfer duration; TST, time span before blood transfusion; LOS, length of stay

The IHTD did not decrease in the with-feedback group. The proportion of patients receiving blood transfusion before transfer increased from 42.31 to 73.24% (*p* = 0.010). The TSBT decreased significantly from 101.5 min to 88.0 min (*p* = 0.046). There was no difference in crude mortality, length of stay, and intensive care unit days between the two groups.

Regular feedback and ISS were factors associated with the time span before PRBC transfusion in univariate analysis, whereas regular feedback was independently associated with the time span before blood transfusion (β − 31.80; SE: 15.14) in the multivariable regression model. (Table 2) Regular feedback, ISS, and shock index ≥0.9 in regional hospital ED were factors associated with blood transfusion before transfer. Regular feedback was independently associated with blood transfusion before transfer in the multivariable regression model (odds ratio [OR]: 2.75; 95% confidence interval [CI]: 1.01–7.52; *p* = 0.049). (Table 3).

**Table 2:** Univariate and multivariate analysis for factors affecting time span before blood transfusion (minutes)

	Univariate model		Multivariable model ^a^	
Variables	β ± SE	*p*-value	β ± SE	*p*-value
Feedback: with vs. without	−36.94 ± 15.13	0.016	−31.80 ± 15.14	0.038
Age: ≥45 vs. < 45	4.74 ± 13.83	0.733		
Male vs. female	19.12 ± 16.38	0.246		
ISS	−1.20 ± 0.52	0.023	−1.01 ± 0.52	0.053
Pre-transfer CPR	−32.48 ± 26.49	0.223		
Shock index: ≥0.9 vs. < 0.9	−25.11 ± 14.16	0.080		

^a^Multivariate linear regression analysis of variables (intervention variable & p-value <  0.05 in univariate linear regression). ISS, injury severity score; CPR, cardiopulmonary resuscitation

**Table 3:** Univariate and multivariate analysis for factors affecting blood transfusion before transfer

Variables	Crude OR (95% CI)	*p*-value	Adjusted OR ^a^ (95% CI)	*p*-value
Feedback: with vs. without	3.73 (1.46–9.54)	0.006	2.75 (1.01–7.52)	0.049
Age: ≥ 45 vs. < 45	0.85 (0.37–1.97)	0.709		
Male vs. female	1.38 (0.52–3.67)	0.513		
ISS	1.05 (1.01–1.09)	0.023	1.04 (1.00–1.08)	0.088
Pre-transfer CPR	1.38 (0.25–7.52)	0.710		
Shock index: ≥0.9 vs. < 0.9	4.80 (1.74–13.25)	0.002	3.55 (1.23–10.23)	0.019

^a^Multivariate logistic regression analysis of variables (intervention variable and *p*-value < 0.05 in univariate logistic regression). ISS, injury severity score; CPR, cardiopulmonary resuscitation

In univariate analysis, ISS, cardiopulmonary resuscitation before transfer, GCS ≤ 8 and transfusion of PRBC ≥12 units were factors associated with a higher mortality rate. After adjustment for ISS, CPR before transfer, massive transfusion of PRBC, and regular feedback was associated with marginally decrease mortality risk (aOR: 0.43; 95% CI: 0.17–1.09; *p* = 0.076). (Table 4).

**Table 4:** Univariate and multivariate analysis for factors predicting mortality

Variables	Crude OR (95% CI)	*p*-value	Adjusted OR ^a^ (95% CI)	*p*-value
Feedback: with vs. without	0.66 (0.31–1.39)	0.272	0.43 (0.17–1.09)	0.076
Age: ≥ 45 vs. < 45	1.16 (0.55–2.45)	0.703		
Male vs. female	1.75 (0.71–4.32)	0.223		
ISS	1.06 (1.03–1.09)	< 0.001	1.03 (0.99–1.07)	0.079
Pre-transfer CPR	6.03 (1.53–23.84)	0.010	2.61 (0.52–13.15)	0.245
RBC: ≥12 U vs. < 12 U	5.63 (2.52–12.56)	< 0.001	3.32 (1.13–9.79)	0.030
GCS: ≤ 8 vs. > 8	7.27 (3.23–16.37)	< 0.001	4.90 (2.03–11.85)	< 0.001
Shock index: ≥0.9 vs. < 0.9	1.51 (0.68–3.36)	0.313		

^a^Multivariate logistic regression analysis of variables (intervention variable and *p*-value < 0.05 in univariate logistic regression). ISS, injury severity score; CPR, cardiopulmonary resuscitation; RBC, red blood cell

## Discussion

**Fig. 1:**
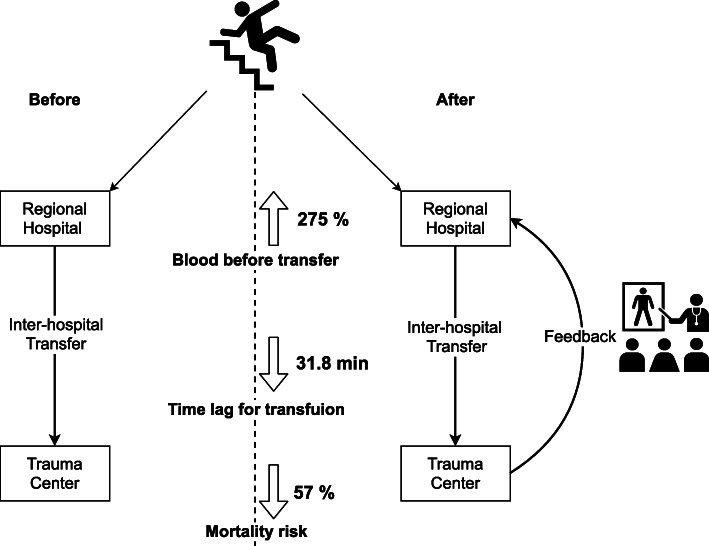
Illustration of the effect of inter-hospital feedback

Our study demonstrated that regular feedback on inter-hospital transfer improved the TSBT on initial resuscitation. Patients with major trauma were more likely to be transfused with PRBCs upon arrival at the emergency department of a regional hospital when the feedback on inter-hospital transfer was performed on a monthly basis. Although it is not statistically significant, the monthly feedback might contribute to decreased mortality risk. (Fig. 1).

Undertriage of major trauma patients is a common issue, and inter-hospital transfer remains a great challenge for the trauma system [8, 11–13]. The published literature focuses mainly on causes and outcomes of undertriage [11, 14, 15]. Building an organized regional trauma system warrants a large amount of resources and may not guarantee rapid inter-hospital transfer [8, 9, 16]. The rural trauma team development course has been reported to decrease the time for patient transfer; however, the risk of death did not reduce as per expectations [10, 17, 18]. To the best of our knowledge, ours is the first study to demonstrate that regular feedback on inter-hospital transfer might contribute to the reduction of mortality risk.

Hemorrhage is the main cause of preventable death in trauma patients [19–21]. Early blood transfusion serves as a bridge to definitive hemostasis procedures so as to reduce the risk of mortality [22]. In fact, our feedback for regional hospitals emphasizes not only the importance of early blood transfusion, but also the transportation of blood components. In a bid to find a comprehensive solution, we break these issues down into small steps and optimize each of them. We have set up a protocol for early notification of blood banks for O+ blood preparation and for the personnel for blood component transportation from the blood bank to the emergency department or the operating room. Our experiences were shared with the regional hospitals, which explains why the mortality risk was decreased in our study, but not in other studies [10, 17].

The context of feedback was not only lectures or presentations on the outcomes of the transferred patients, but also communication and interaction of emergency department physicians and trauma surgeons about the transfer details. The feedback was meant to be innovative and oriented towards problem-solving instead of fault-finding and anxiety-provoking. In the process of monthly feedback, we acknowledged that one-way feedback from the tertiary trauma center focusing on the backend processing more often becomes captious or hypercritical than constructive in terms of a rapidly responsive and effective trauma system. Breaking down the transfer issue into small steps highlighted the emerging problem and enhanced the will for cooperation especially when the triage took the right measures towards initial resuscitation. Interaction from both sides helped emergency physicians and trauma surgeons comprehend patient evaluation at the scene and the rationale of decision-making on hemostasis, thus, compensating for the gap in judgment.

Our study had some limitations. Firstly, selection bias was inevitable because of the retrospective nature of the study. Secondly, the small number of study cohorts in a single tertiary center was a disadvantage. Thirdly, the shortage of medical personnel at the emergency department in the regional hospital might have been overlooked. Fourthly, bleeding is a major risk factor of mortality. In our study, patients requiring blood transfusion ≥12 U had a significantly high risk of mortality. However, the transfusion amount was not affected by the patient’s injury only. We set up a massive transfusion protocol (MTP) since 2015 that may improve transfusion intensity and quality. This may have overestimated the protective effect of inter-hospital feedback. Lastly, since inter-hospital transfer occurred in severely injured yet salvageable patients, patients presenting with cardiopulmonary collapse and dismal outcomes might have been neglected. Further investigation of a larger population among different tertiary centers is necessary to consolidate our conclusions.

## Conclusions

Regular feedback on inter-hospital referrals improved quality by facilitating blood transfusion before patient transfer. The impact on mortality risk needs further validation.

## Data Availability

The datasets used and analyzed during the current study are available from the corresponding author on reasonable request.

## Abbreviations

IHTD: inter-hospital transfer duration
ED: emergency department
TSBT: time span before blood transfusion
PRBCs: packed red blood cells
ISS: injury severity score
CT: computed tomography
